# Predictive value of lymphocyte‐to‐monocyte ratio in critically Ill patients with atrial fibrillation: A propensity score matching analysis

**DOI:** 10.1002/jcla.24217

**Published:** 2021-12-30

**Authors:** Yue Yu, Suyu Wang, Pei Wang, Qiumeng Xu, Yufeng Zhang, Jian Xiao, Xiaofei Xue, Qian Yang, Wang Xi, Junnan Wang, Renhong Huang, Meiyun Liu, Zhinong Wang

**Affiliations:** ^1^ Department of Cardiothoracic Surgery Changzheng Hospital Naval Medical University Shanghai China; ^2^ Department of Orthopaedics Changzheng Hospital Naval Medical University Shanghai China; ^3^ Department of General Surgery Comprehensive Breast Health Center Ruijin Hospital Shanghai Jiaotong University School of Medicine Shanghai China; ^4^ Department of Anesthesiology Shanghai Pulmonary Hospital Tongji University School of Medicine Shanghai China

**Keywords:** atrial fibrillation, inflammation, lymphocyte‐to‐monocyte ratio, MIMIC‐III database, mortality, prognostic biomarker, regression analysis

## Abstract

**Background:**

Inflammation plays a key role in the initiation and progression of atrial fibrillation (AF). Lymphocyte‐to‐monocyte ratio (LMR) has been proved to be a reliable predictor of many inflammation‐associated diseases, but little data are available on the relationship between LMR and AF. We aimed to evaluate the predictive value of LMR in predicting all‐cause mortality among AF patients.

**Methods:**

Data of patients diagnosed with AF were retrieved from the Medical Information Mart for Intensive Care‐III (MIMIC‐III) database. X‐tile analysis was used to calculate the optimal cutoff value for LMR. The Cox regression model was used to assess the association of LMR and 28‐day, 90‐day, and 1‐year mortality. Additionally, a propensity score matching (PSM) method was performed to minimize the impact of potential confounders.

**Results:**

A total of 3567 patients hospitalized with AF were enrolled in this study. The X‐tile software indicated that the optimal cutoff value of LMR was 2.67. A total of 1127 pairs were generated, and all the covariates were well balanced after PSM. The Cox proportional‐hazards model showed that patients with the low LMR (≤2.67) had a higher 1‐year all‐cause mortality than those with the high LMR (>2.67) in the study cohort before PSM (HR = 1.640, 95% CI: 1.437–1.872, *p *< 0.001) and after PSM (HR = 1.279, 95% CI: 1.094–1.495, *p *= 0.002). The multivariable Cox regression analysis for 28‐day and 90‐day mortality yielded similar results.

**Conclusions:**

The lower LMR (≤2.67) was associated with a higher risk of 28‐day, 90‐day, and 1‐year all‐cause mortality, which might serve as an independent predictor in AF patients.

## Graphical abstract

Our study was the first to investigate the association between admission lymphocyte‐to‐monocyte ratio (LMR) in the peripheral blood and risk of death among critically ill patients with atrial fibrillation with a 1‐year follow‐up. We analyzed LMR as a continuous variable and used restricted cubic spline regression analysis to estimate the association between LMR and its hazard ratios. In addition, we analyzed LMR as a categorical variable and performed the Cox proportional‐hazards regression model to assess the association of LMR and 28‐day, 90‐day, and 1‐year mortality. A propensity score matching method was performed to minimize the impact of potential confounders.

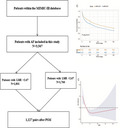

## 1 INTRODUCTION

Atrial fibrillation (AF) is the most common sustained and supraventricular arrhythmia, characterized by uncoordinated atrial electrical activation and consequently ineffective atrial contraction.^1^ AF is associated with substantial morbidity and mortality, thus posing a significant burden to patients, physicians, and healthcare systems globally.^2^ Preventing AF recurrence (via rhythm control) and detrimental complications (via rate control and antithrombotic therapies) are current therapeutic strategies for AF patients.^3^ The pathophysiology of AF is complex and incompletely understood. Emerging evidence suggests that the roles of activated inflammatory cells and mediators in cardiac tissue and circulatory system have been implicated in various AF‐related pathological mechanisms.^4^, ^5^

The lymphocyte‐to‐monocyte ratio (LMR), comprised of the ratio of white blood cell (WBC) subgroups, has been proved to be a novel inflammatory marker for lots of cardiovascular diseases, such as acute type A aortic dissection (AAAD),^6^ ST‐elevated myocardial infarction (STEMI),^7^ heart failure,^8^ acute pulmonary embolism,^9^ and carotid artery stenosis.^10^ Several histological studies of AF found that increased infiltration of inflammatory cells, such as lymphocytes and monocytes, in the atrial myocardium or appendage tissues.^11^, ^12^, ^13^ Another study demonstrated that a higher percentage of activated T lymphocytes was observed in the peripheral blood of patients with paroxysmal or persistent AF.^14^ Furthermore, monocyte infiltration in the left atria was reported to be associated with AF‐related thromboembolic events.^15^, ^16^ Nevertheless, to the best of our knowledge, there is almost no study investigating the association between LMR in the peripheral blood and the survival of AF patients.

In the present study, we intended to investigate whether there was a relationship between LMR and prognosis in critically ill patients with AF by utilizing the Medical Information Mart for Intensive Care‐III (MIMIC‐III) database. This research was conducted consistent with the requirements of the STrengthening the Reporting of OBservational studies in Epidemiology (STROBE) statement.^17^

## 2 MATERIALS AND METHODS

### 2.1 Study design and data resource

We conducted a longitudinal, single‐center retrospective cohort study with all the relevant data collected from the MIMIC‐III database based on the methods used in our previous studies.^18^, ^19^, ^20^ The MIMIC‐III database is an open and freely accessible database collecting data from over 50,000 critically ill patients at the Beth Israel Deaconess Medical Center (BIDMC) in Boston from 2001 to 2012.^21^ The MIMIC‐III database documents contained comprehensive and high‐quality data from hospital monitoring systems and bedside monitoring systems. International Classification of Diseases, Ninth Revision (ICD‐9) code was documented for specific diseases by hospital staff on patient discharge. We obtained permission to access the dataset after passing the “Protecting Human Research Participants” exam (authorization code: 33281932). The establishment of the MIMIC‐III database was approved by the Institutional Review Boards of the Massachusetts Institute of Technology (Cambridge, MA, USA) and BIDMC, and consent was obtained for the original data collection. Therefore, the ethics approval statement and the requirement for informed consent were waived. In summary, this study conformed to the provisions of the Declaration of Helsinki (as revised in Edinburgh 2000).

### 2.2 Patient selection

We included all intensive care unit (ICU) patients (aged ≥ 18 years) in the database with the primary diagnosis of AF using the ICD‐9 diagnosis code (ICD‐9 code of AF = 42731). Only the data of each patient's first ICU admission were used in this study. Patients were excluded if they had (1) a secondary diagnosis of inflammatory, hematological or autoimmune diseases, sepsis, or malignant tumors; (2) incomplete follow‐up information; (3) a length of stay in the ICU less than 24 hours; (4) incomplete or unobtainable data of measured lymphocyte or monocyte count during the first 24‐hour admission; or (5) more than 10% of individual data missing.

### 2.3 Data extraction and study outcomes

Structured query language with PostgreSQL (version 9.4.6, www.postgresql.org) was used to extract data on demographics, vital signs, laboratory tests, scoring systems, and treatment information from the database. Baseline demographic variables included age, sex, and current smoking status. We extracted data on the following comorbidities: coronary artery disease (CAD), congestive heart failure, hypertension, chronic obstructive pulmonary disease (COPD), stroke, transient ischemic attack (TIA), diabetes mellitus (DM), dyslipidemia, anemia, chronic kidney disease, chronic liver disease, and sleep apnea. Vital signs on admission included heart rate, respiratory rate, systolic blood pressure (SBP), diastolic blood pressure (DBP), and mean blood pressure (MBP). Laboratory‐based data included WBC, neutrophil, lymphocyte, platelet, monocyte, hematocrit, hemoglobin, red blood cell distribution width (RDW), albumin, blood urea nitrogen (BUN), creatinine, glucose, total calcium (tCa), potassium, sodium, chloride, magnesium, prothrombin time (PT), partial thromboplastin time (PTT), and international normalized ratio (INR). If participants underwent more than one laboratory test during their hospitalization, only the initial test results were included for further analysis. In terms of scoring systems, the Simplified Acute Physiology Score II (SAPS II) and the Sequential Organ Failure Assessment (SOFA) were extracted from the database. Additionally, treatment information data included mechanical ventilation, renal replacement treatment, appendage closure, coronary artery bypass grafting (CABG), valvular surgery, and in‐hospital medication administration (antiarrhythmic agents, antiplatelet agents, warfarin, and beta‐blocker).

Our primary study outcome was 1‐year all‐cause mortality. The secondary outcomes included 28‐day and 90‐day all‐cause mortality.

### 2.4 Definition, calculation, and identification of cutoff values for LMR

Lymphocyte‐to‐monocyte ratio was calculated in the formulate: lymphocyte counts divided by monocyte counts on admission. LMR, as a continuous variable, was dichotomized via the X‐tile software (version 3.6.1; Yale University, New Haven, CT, USA) based on the maximal log‐rank chi‐square value, which represented the greatest group difference in outcome probability.^22^ In addition, normal ranges of lymphocyte and monocyte counts in the peripheral blood were defined as between 0.8 × 10^9^/L and 4.0 × 10^9^/L, and between 0.12 × 10^9^/L and 0.8 × 10^9^/L, respectively.

### 2.5 Management of missing data

To reduce bias due to missing data, variables with more than 20% missing values were excluded from the study. Correspondingly, variables with less than 20% missing values were handled using multivariable imputation.^23^ Variables for which multivariable imputation was adopted included RDW, BUN, tCa, chloride, PT, PTT, and INR.

### 2.6 Propensity score matching

Propensity score matching (PSM) analysis was used to minimize the effect of potential confounders. Baseline characteristics (age, sex, current smoking status, admission type, CAD, congestive heart failure, hypertension, COPD, stroke, TIA, DM, dyslipidemia, anemia, chronic kidney disease, chronic liver disease, sleep apnea, SBP, DBP, MBP, heart rate, respiratory rate, WBC, neutrophil, platelet, hematocrit, hemoglobin, RDW, albumin, BUN, creatinine, tCa, potassium, sodium, chloride, magnesium, PT, PTT, INR, SOFA, SAPS II, mechanical ventilation, renal replacement treatment, appendage closure, CABG, valvular surgery, and in‐hospital medication administration) were incorporated in the propensity score analysis. We did not include lymphocyte and monocyte counts in the PSM analysis to avoid influence on the value of LMR. A logistic regression model was constructed to calculate and assign each patient a propensity score, which was defined as the likelihood of being exposed to an intervention given that the status of a particular patient's measured prognostic factors.^24^, ^25^ Next, 1:1 matching (LMR ≤ 2.67 *vs*. LMR > 2.67) without replacement was performed using a nearest neighbor matching algorithm, with a fixed caliper width of 0.05.^26^

### 2.7 Statistical analysis

The data distribution was examined using the Kolmogorov‐Smirnov test. Categorical variables are presented as total number and percentage, and continuous variables as mean (standardized differences [SD]) or median (interquartile range [IQR]). Baseline characteristics of enrolled participants were presented by using either Pearson's chi‐square test, Fisher's exact test, Student *t* test, or Mann–Whitney *U* test as appropriate.

The unadjusted survival curves were plotted by the Kaplan–Meier method and compared using the log‐rank test. Moreover, Cox proportional‐hazards analysis was performed to examine the relationship between LMR and each study endpoint. Multivariable Cox regression Model 1 was adjusted for age and sex. Multivariable Cox regression Model 2 was adjusted for variables with *p *< 0.100 in the univariable Cox analysis. The results of Cox regression models are presented as hazard ratios (HRs) and 95% confidence intervals (CIs). The LMR > 2.67 group was taken as the reference group. We also did the subgroup analysis based on lymphocyte and monocyte counts, age, sex, CAD, congestive heart failure, hypertension, COPD, stroke, TIA, DM, dyslipidemia, anemia, chronic kidney disease, chronic liver disease, sleep apnea, mechanical ventilation, CABG, renal replacement treatment, and in‐hospital medication administration. Furthermore, to identify a non‐linear relationship, a smooth curve was then drawn to estimate the relationship between LMR and its HR using restricted cubic spline regression analysis. Two piece‐wise Cox proportional‐hazards models were further performed to demonstrate the saturation effect of LMR on mortality. The inflection point was determined using the recursive method, where the model gave the maximum likelihood. Furthermore, a log‐likelihood ratio test comparing the one‐line linear model with two piece‐wise models was conducted to determine whether the saturation effect existed.

A two‐tailed *p *< 0.050 was considered to be statistically significant. All statistical analyses were conducted using R software (version.3.6.1; The R Project for Statistical Computing, TX, USA; http://www.r‐project.org) and SPSS software (version 22.0; IBM Corporation, St. Louis, Missouri, USA).

## 3 RESULTS

### 3.1 Characteristics of patients

**FIGURE 1:**
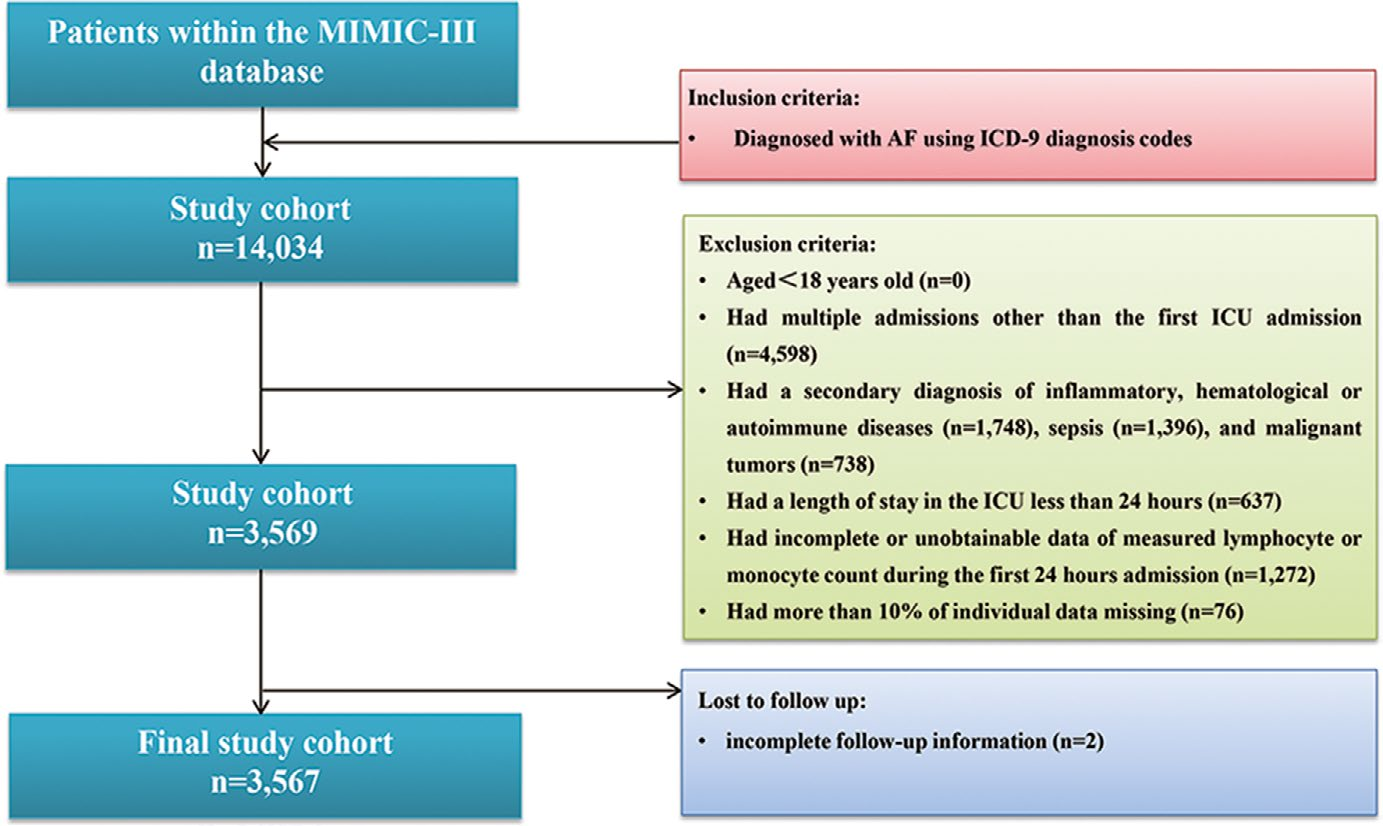
Flow diagram of patient inclusion. MIMIC‐III, Medical Information Mart for Intensive Care‐III; ICU, intensive care unit; ICD‐9, International Classification of Diseases, Ninth Revision

In total, 3567 patients fulfilled the selection criteria and comprised the final study cohort (Figure 1). X‐tile software identified the optimal cutoff value of LMR for 1‐year mortality as 2.67. Therefore, patients were divided into the low LMR group (*n* = 1766) and the high LMR group (*n* = 1801). The baseline characteristics of enrolled patients are briefly summarized in Table 1. Patients with the higher LMR (>2.67) tended to be younger (*p *< 0.001). Regarding comorbidity, patients with the higher LMR (>2.67) were more likely to suffer from CAD (*p *= 0.002), hypertension (*p *< 0.001), stroke (*p *= 0.028), and dyslipidemia (*p *< 0.001). However, patients with the lower LMR (≤2.67) displayed higher WBC (*p *< 0.001), neutrophil (*p *< 0.001), platelet (*p *< 0.001), monocyte (*p *< 0.001), hematocrit (*p *= 0.007), RDW (*p *< 0.001), BUN (*p *< 0.001), creatinine (*p *< 0.001), glucose (*p *< 0.001), PT (*p *= 0.030), INR (*p *= 0.004), SOFA (*p *< 0.001), and SAPS II (*p *< 0.001); they were also more likely to receive renal replacement treatment (*p *< 0.001).

**TABLE 1:** Characteristics of the study patients according to the LMR groups before and after PSM

Characteristics	Before PSM			After PSM		
	LMR > 2.67 (*n* = 1801)	LMR ≤ 2.67 (*n* = 1766)	*p* value	LMR > 2.67 (*n* = 1127)	LMR ≤ 2.67 (*n* = 1127)	*p* value
*Demographics*						
Age, years	75 (66–83)	77 (68–84)	<0.001	77 (67–84)	77 (68–84)	0.975
Sex, male, n (%)	982 (54.5)	1,008 (57.1)	0.125	637 (56.5)	614 (54.5)	0.330
Current smoker, n (%)	843 (46.8)	847 (48)	0.490	520 (46.1)	519 (46.1)	0.966
*Admission type, n (%)*						
Elective	429 (23.8)	272 (15.4)	<0.001	208 (18.5)	213 (18.9)	0.938
Emergency	1,325 (73.6)	1,433 (81.1)		887 (78.7)	884 (78.4)	
Urgent	47 (2.6)	61 (3.5)		32 (2.8)	30 (2.7)	
*Comorbidities, n (%)*						
CAD	877 (48.7)	770 (43.6)	0.002	509 (45.2)	513 (45.5)	0.866
Congestive heart failure	759 (42.1)	911 (51.6)	<0.001	552 (49.0)	544 (48.3)	0.736
Hypertension	989 (54.9)	834 (47.2)	<0.001	579 (51.4)	584 (51.8)	0.833
COPD	223 (12.4)	307 (17.4)	<0.001	166 (14.7)	170 (15.1)	0.813
Stroke	249 (13.8)	201 (11.4)	0.028	145 (12.9)	146 (13.0)	0.950
TIA	50 (2.8)	33 (1.9)	0.072	29 (2.6)	27 (2.4)	0.787
DM	519 (28.8)	504 (28.5)	0.854	326 (28.9)	309 (27.4)	0.426
Dyslipidemia	420 (23.3)	278 (15.7)	<0.001	202 (17.9)	216 (19.2)	0.448
Anemia	451 (25)	480 (27.2)	0.146	297 (26.4)	297 (26.4)	1.000
Chronic kidney disease	248 (13.8)	323 (18.3)	<0.001	183 (16.2)	167 (14.8)	0.352
Chronic liver disease	31 (1.7)	49 (2.8)	0.034	24 (2.1)	25 (2.2)	0.885
Sleep apnea	90 (5.0)	79 (4.5)	0.462	61 (5.4)	49 (4.3)	0.241
*Vital signs*						
SBP, mmHg	113.2 (104.5–125)	114.3 (105–125.5)	0.380	113.2 (104.2–125.2)	114.1 (104.7–125.4)	0.604
DBP, mmHg	56.7 (51.2–63.1)	57.0 (51.4–63.4)	0.124	56.8 (51.5–63.1)	56.6 (51.3–63.2)	0.539
MBP, mmHg	73.6 (68.3–80.6)	74.3 (68.8–81.2)	0.149	74.0 (68.6–80.5)	74.0 (68.4–80.7)	0.729
HR, beats/min	83.6 (73.3–93.8)	82.7 (73.2–93.5)	0.518	83.1 (73.0–93.5)	82.8 (72.9–93.3)	0.577
RR, beats/min	18.5 (16.4–21.3)	18.5 (16.2–21.4)	0.846	18.5 (16.3–21.3)	18.4 (16.1–21.2)	0.796
*Laboratory‐based data*						
WBC, 10^9^/L	10.5 (8.0–14.0)	11.6 (8.7–15.6)	<0.001	10.4 (7.8–13.8)	10.6 (8.1–14.2)	0.122
Neutrophil, 10^9^/L	7.6 (5.6–10.6)	9.5 (6.9–13.0)	<0.001	8.0 (5.8–11.1)	8.4 (6.3–11.4)	0.065
Lymphocyte, 10^9^/L	1.8 (1.2–2.7)	0.9 (0.6–1.4)	<0.001	1.5 (1.1–2.1)	1.0 (0.6–1.5)	<0.001
Platelet, 10^9^/L	187.0 (143.0–242.0)	196.0 (144.0–264.0)	<0.001	191.0 (144.0–247.5)	190.0 (141.0–249.0)	0.514
Monocyte, 10^9^/L	0.4 (0.3–0.6)	0.6 (0.4–0.9)	<0.001	0.4 (0.2–0.5)	0.6 (0.4–0.9)	<0.001
Hematocrit, %	30.6 (27.1–34.9)	31.4 (27.5–35.0)	0.007	30.9 (27.5–35.1)	31.1 (27.3–34.8)	0.805
Hemoglobin, g/dL	10.4 (9.2–11.8)	10.5 (9.3–11.8)	0.182	10.5 (9.3–11.9)	10.5 (9.2–11.7)	0.716
RDW, %	14.4 (13.6–15.5)	14.7 (13.8–16.1)	<0.001	14.6 (13.7–15.9)	14.6 (13.7–15.8)	0.576
Albumin, mg/dL	3.4 (2.9–3.9)	3.2 (2.7–3.7)	<0.001	3.3 (2.8–3.7)	3.3 (2.8–3.8)	0.270
BUN, mg/dL	21.0 (15.0–31.0)	25.0 (17.0–40.0)	<0.001	23.0 (17.0–36.0)	23.0 (16.0–35.0)	0.539
Creatinine, mg/dL	1.0 (0.7–1.3)	1.1 (0.8–1.7)	<0.001	1.0 (0.8–1.5)	1.0 (0.8–1.4)	0.654
Glucose, mg/dL	124.0 (103.0–150.0)	129.0 (107.0–161.0)	<0.001	126.0 (105.0–155.0)	127.0 (107.0–157.0)	0.807
tCa, mg/dL	8.4 (8.0–8.8)	8.4 (7.9–8.8)	0.532	8.4 (7.9–8.8)	8.4 (7.9–8.8)	0.933
Potassium, mmol/L	4.2 (3.8–4.6)	4.2 (3.8–4.6)	0.179	4.2 (3.8–4.6)	4.2 (3.8–4.6)	0.774
Sodium, mmol/L	139.0 (137.0–142.0)	139.0 (136.0–141.0)	<0.001	139.0 (137.0–141.0)	139.0 (137.0–141.0)	0.893
Chloride, mmol/L	108.0 (104.0–111.0)	107.0 (103.0–110.0)	<0.001	107.0 (103.0–111.0)	107.0 (103.3–111.0)	0.323
Magnesium, mmol/L	2.0 (1.8–2.3)	2.0 (1.8–2.3)	0.520	2.0 (1.8–2.3)	2.0 (1.8–2.3)	0.764
PT, s	15.7 (14–18.5)	15.8 (14.1–19.2)	0.030	15.8 (14–19.1)	15.6 (14.0–18.6)	0.311
PTT, s	37.4 (30.1–53.1)	37.2 (30.1–55.4)	0.282	37.3 (30.1–54)	37 (30.2–55.0)	0.564
INR, s	1.5 (1.2–1.9)	1.5 (1.3–2.0)	0.004	1.5 (1.2–2.0)	1.5 (1.3–1.9)	0.253
*Scoring system*						
SOFA	4.0 (2.0–6.0)	4.0 (2.0–6.0)	<0.001	4.0 (2.0–6.0)	4.0 (2.0–6.0)	0.399
SAPS II	35.0 (29.0–43.0)	38.0 (31.0–46.0)	<0.001	36.0 (30.0–45.0)	37.0 (30.0–44.0)	0.619
*Treatment information, n (%)*						
Mechanical ventilation	1,074 (59.6)	1,000 (56.6)	0.069	625 (55.5)	627 (55.6)	0.932
Renal replacement therapy	23 (1.3)	50 (2.8)	0.001	22 (2.0)	18 (1.6)	0.523
Appendage closure	22 (1.2)	10 (0.6)	0.038	6 (0.5)	8 (0.7)	0.592
CABG	508 (28.2)	349 (19.8)	<0.001	251 (22.3)	257 (22.8)	0.762
Valvular surgery	57 (3.2)	39 (2.2)	0.078	25 (2.2)	25 (2.2)	1.000
*In‐hospital medication, n (%)*						
Antiarrhythmic agents	1,653 (91.8)	1,570 (88.9)	0.004	1018 (90.3)	1030 (91.4)	0.380
Antiplatelet agents	1,529 (84.9)	1,402 (79.4)	<0.001	915 (81.2)	912 (80.9)	0.872
Warfarin	843 (46.8)	721 (40.8)	<0.001	480 (42.6)	497 (44.1)	0.470
Beta‐blocker	1,401 (77.8)	1,266 (71.7)	<0.001	833 (73.9)	847 (75.2)	0.498

Note

BUN, blood urea nitrogen; CABG, coronary artery bypass grafting; CAD, coronary artery disease; COPD, chronic obstructive pulmonary disease; DBP, diastolic blood pressure; DM, diabetes mellitus; INR, international normalized ratio; LMR, lymphocyte‐to‐monocyte ratio; LMR, lymphocyte‐to‐monocyte ratio; MBP, mean blood pressure; PSM, propensity score matching; PT, prothrombin time; PTT, partial thromboplastin time; RDW, red cell distribution width; RR, respiratory rate; SAPS II, Simplified Acute Physiology Score II; SBP, systolic blood pressure; SOFA, Sequential Organ Failure Assessment; tCA, total calcium; TIA, transient ischemic attacks; WBC, white blood cell.

### 3.2 Prognostic significance of LMR before PSM

**FIGURE 2:**
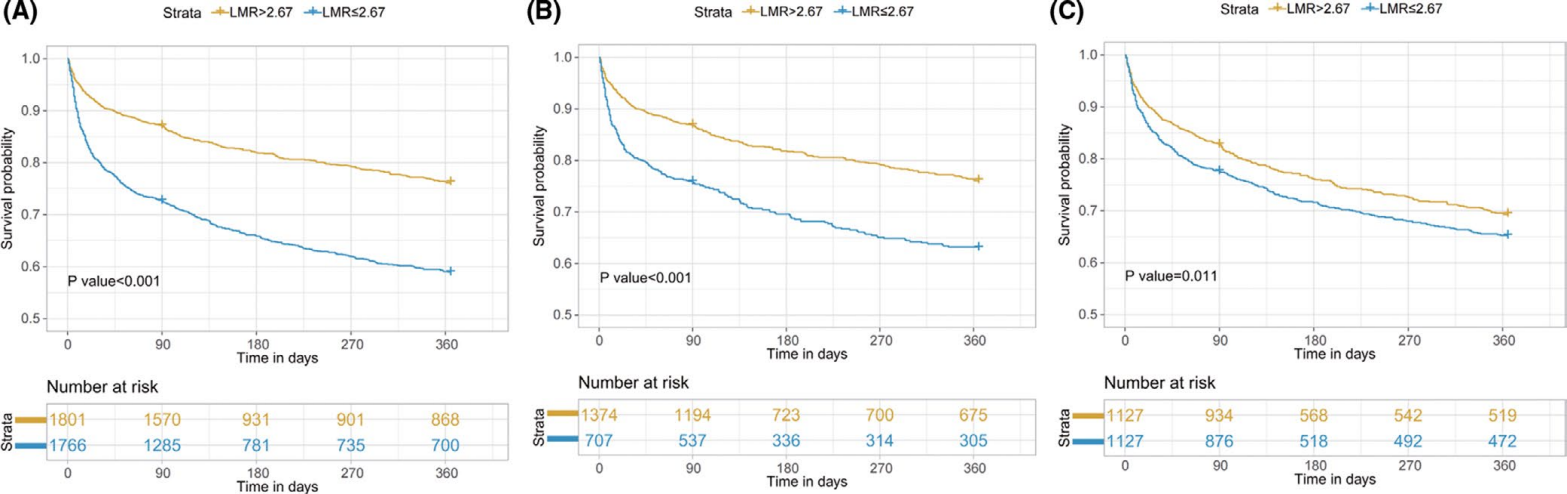
Kaplan–Meier survival analysis plot for 1‐year survival. A significantly lower 1‐year survival rate can be observed in the lower LMR group in patients before PSM (A), patients with normal lymphocyte and monocyte counts (B), and patients after PSM (C). LMR, lymphocyte‐to‐monocyte ratio; PSM, propensity score matching

Among the 3567 AF patients included, 13.9% (495/3567) died during the first 28 days, 20.1% (717/3567) died during the first 90 days, and 28.1% (1004/3567) died during the 1‐year follow‐up period. Kaplan–Meier curves for all‐cause death according to the LMR groups are shown in Figure 2A. The curves of the LMR groups differed significantly, and patients in the low LMR group had a higher cumulative incidence of mortality (log‐rank test: *p *< 0.001).

The results of the univariable and multivariable Cox regression analyses are summarized in Table 2 and Tables S1–3. A univariable Cox regression analysis was conducted to select the variables with *p *< 0.100, and age, gender, CAD, congestive heart failure, hypertension, COPD, stroke, dyslipidemia, chronic kidney disease, chronic liver disease, sleep apnea, mechanical ventilation, renal replacement treatment, appendage closure, CABG, valvular surgery, antiarrhythmic, antiplatelet agents, warfarin, and beta‐blocker were selected and incorporated into the multivariable Cox regression model. Multivariable Cox regression analysis showed that patients with the LMR ≤ 2.67 had significantly higher 1‐year mortality compared to patients with the LMR > 2.67 (Model 1: HR = 1.950, 95% CI: 1.713–2.220, *p *< 0.001; Model 2: HR = 1.640, 95% CI: 1.437–1.872, *p *< 0.001). The multivariable analysis for 28‐day and 90‐day mortality yielded similar results.

**TABLE 2:** Outcomes of patients before and after PSM and patients with normal lymphocyte and monocyte counts

	Unadjusted model		Adjusted model 1		Adjusted model 2	
	HR (95% CI)	*p* value	HR (95% CI)	*p* value	HR (95% CI)	*p* value
*Before PSM*						
28‐day mortality	2.434 (2.011–2.947)	<0.001	2.295 (1.895–2.780)	<0.001	1.816 (1.494–2.208)	<0.001
90‐day mortality	2.312 (1.978–2.704)	<0.001	2.173 (1.857–2.542)	<0.001	1.784 (1.521–2.092)	<0.001
1‐year mortality	2.059 (1.809–2.343)	<0.001	1.950 (1.713–2.220)	<0.001	1.640 (1.437–1.872)	<0.001
*After PSM*						
28‐day mortality	1.403 (1.111–1.771)	0.004	1.396 (1.106–1.763)	0.005	1.447 (1.145–1.830)	0.002
90‐day mortality	1.341 (1.113–1.617)	0.002	1.344 (1.115–1.619)	0.002	1.416 (1.174–1.708)	<0.001
1‐year mortality	1.223 (1.047–1.429)	0.011	1.217 (1.042–1.422)	0.013	1.279 (1.094–1.495)	0.002
*Normal lymphocytes and monocytes group*						
28‐day mortality	2.215 (1.730–2.836)	<0.001	2.095 (1.635–2.685)	<0.001	1.755 (1.360–2.266)	<0.001
90‐day mortality	1.973 (1.601–2.432)	<0.001	1.852 (1.501–2.284)	<0.001	1.548 (1.249–1.920)	<0.001
1‐year mortality	1.781 (1.496–2.121)	<0.001	1.674 (1.405–1.994)	<0.001	1.442 (1.205–1.724)	<0.001

Note

CABG, coronary artery bypass grafting; CAD, coronary artery disease; CI, confidential interval; COPD, chronic obstructive pulmonary disease; HR, hazard ratio; LMR, lymphocyte‐to‐monocyte ratio; PSM, propensity score matching.

^a^ Adjusted model 1 was adjusted by age and sex.

^b^ Adjusted model 2 was adjusted by age, gender, CAD, congestive heart failure, hypertension, COPD, stroke, dyslipidemia, chronic kidney disease, chronic liver disease, sleep apnea, mechanical ventilation, renal replacement treatment, appendage closure, CABG, valvular surgery, antiarrhythmic, antiplatelet agents, warfarin, beta‐blocker.

^c^ The LMR >2.67 group was taken as the reference group.

### 3.3 Prognostic significance of LMR after PSM

In total, 1127 pairs of propensity score‐matched patients were generated after using a 1:1 ratio PSM analysis to balance the potential confounders. The patients’ baseline characteristics after PSM are illustrated in Table 1. PSM was effective in controlling the covariate imbalance. A total of 50 covariates were well balanced (*p *> 0.050) between the two groups (LMR ≤ 2.67 *vs*. LMR > 2.67) after PSM analysis.

Among the 2254 AF patients included after PSM, 12.9% (290/2254) died during the first 28 days, 19.9% (448/2254) died during the first 90 days, and 28.3% (638/2254) died during the 1‐year follow‐up period. Additionally, the survival curves (Figure 2C) comparing the two groups showed that patients with the LMR≤2.67 still had a lower 1‐year survival rate compared to those with the LMR > 2.67 (log‐rank test: *p *= 0.011).

The results of the univariable and multivariable Cox analyses are summarized in Table 2 and Tables S4–6. In the multivariable Cox regression analysis, patients with the LMR ≤ 2.67 had significantly higher 1‐year mortality compared to those with the LMR > 2.67 (Model 1: HR = 1.217, 95% CI: 1.042–1.422, *p *= 0.013; Model 2: HR = 1.279, 95% CI: 1.094–1.495, *p *= 0.002). The multivariable analysis for 28‐day and 90‐day mortality yielded similar results.

### 3.4 Prognostic significance of LMR in patients with normal lymphocyte and monocyte counts

Considering a reduced lymphocyte count or elevated monocyte count might cause a lower LMR, which could influence the study results independently, the correlation between LMR and mortality was also analyzed in AF patients with normal lymphocyte and monocyte counts. Kaplan‐Meier curves for all‐cause death according to the LMR groups are shown in Figure 2B. The curves of the LMR groups differed significantly, and patients in the low LMR group had a higher cumulative incidence of mortality (log‐rank test: *p *< 0.001). The results of multivariable Cox regression analysis showed that an LMR ≤ 2.67 remained to be an independent prognostic indicator of higher 1‐year mortality (Model 1: HR = 1.674, 95% CI: 1.405–1.994, *p *< 0.001; Model 2: HR = 1.442, 95% CI: 1.205–1.724, *p *< 0.001) (Table 2 and Tables S7–9). The multivariable analysis for 28‐day and 90‐day mortality yielded similar results.

### 3.5 Subgroup analysis

To further validate the robustness of our findings, we performed subgroup analyses to assess the association between LMR and 28‐day, 90‐day, and 1‐year all‐cause mortality. For 1‐year mortality, subgroup analyses showed the lower LMR was also associated with deteriorative mortality in most strata except in patients with chronic liver disease (*p *= 0.065), sleep apnea (*p *= 0.095), or receiving renal replacement treatment (*p *= 0.077) or CABG (*p *= 0.156) (Figure S3). The results for 28‐day and 90‐day mortality were shown in Figures S1–2.

### 3.6 Restricted cubic spline analysis

**FIGURE 3:**
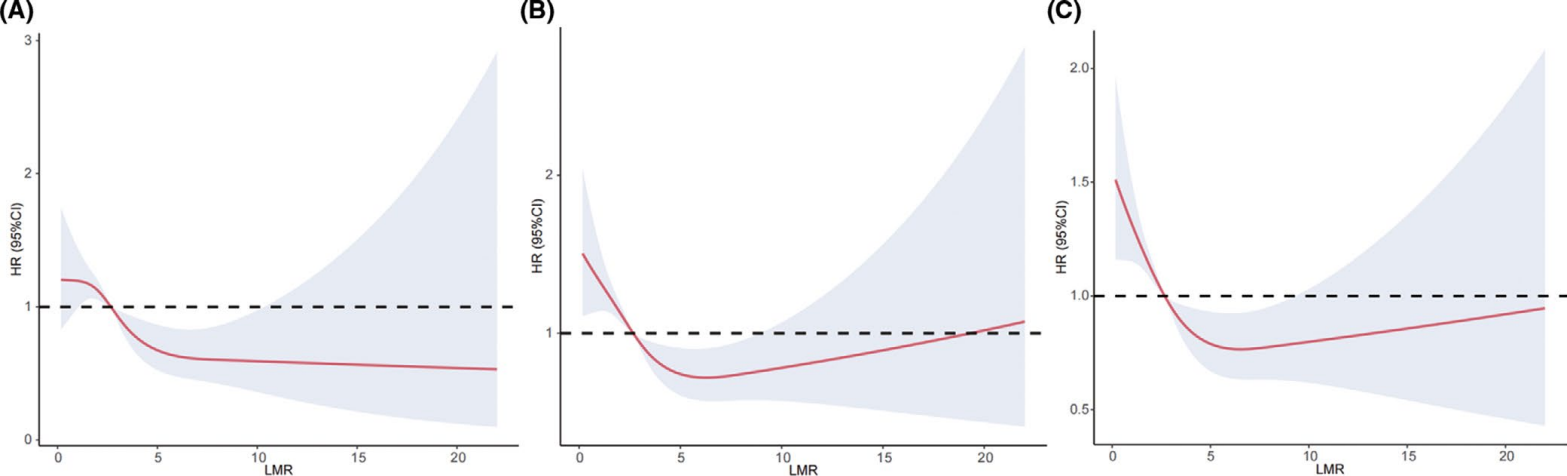
Restricted cubic spline fitting for the association between LMR levels with the HR of LMR for 28‐day (A), 90‐day (B), 1‐year (C) mortality. HRs were evaluated by setting the LMR value=2.67 as reference based on multivariable Cox proportional regression model adjusted by age, gender, coronary artery disease, congestive heart failure, hypertension, COPD, stroke, dyslipidemia, chronic kidney disease, chronic liver disease, sleep apnea, mechanical ventilation, renal replacement treatment, appendage closure, CABG, valvular surgery, antiarrhythmic, antiplatelet agents, warfarin, betablocker. The shaded area represents the 95% CI. CABG, coronary artery bypass grafting; CI, confidential interval; COPD, chronic obstructive pulmonary disease; LMR, lymphocyte‐to‐monocyte ratio

Restricted cubic spline analyses showed an L‐shaped relationship between LMR and the risk of mortality (Figure 3). The logarithm likelihood ratio test revealed the non‐linear relationship between LMR and 90‐day or 1‐year mortality with a point of inflection at 5.33 and 5.50, respectively, indicating a saturation effect in the relationship between LMR and 90‐day or 1‐year mortality (two *P* values <0.001; Table S10). For the LMR < 5.33, every 1 increase in LMR was associated with an 18.9% decrease in 90‐day mortality (*p *< 0.001), while for the LMR > 5.33, every 1 increase in LMR was associated with a 2.1% increase in 90‐day mortality (*p *= 0.519). For an LMR < 5.50, every 1 increase in LMR was associated with a 16.7% decrease in 1‐year mortality (*p *< 0.001), while for an LMR > 5.50, every 1 increase in LMR was associated with a 1.9% increase in 1‐year mortality (*p *= 0.464).

## 4 DISCUSSION

Our study investigated the association between admission LMR in the peripheral blood and risk of death among critically ill patients with AF with a 1‐year follow‐up. Our findings showed that that the lower LMR (≤2.67) was associated with a higher risk of 28‐day, 90‐day, and 1‐year all‐cause mortality and might serve as a reliable predictor of mortality in AF patients. As far as we know, this is the first research to explore the correlation between LMR and mortality of AF patients.

A considerable number of clinical studies have suggested that LMR could serve as an indispensable prognostic predictor in many cardiovascular diseases such as AAAD ^6^, STEMI (7), heart failure (8), acute pulmonary embolism (9), and carotid artery stenosis (10). Moreover, one recent study suggested that a preoperative lower LMR (<3.58) was associated with a higher risk of 4‐year mortality in patients undergoing cardiac surgery.^27^ To date, several circulating blood cell‐based prognostic biomarkers have also been developed to predict clinical outcomes in AF. An elevated neutrophil‐to‐lymphocyte ratio (NLR) before or after catheter ablation was associated with increased AF recurrence after the procedure.^28^, ^29^, ^30^ Gungor et al.^31^ and Saskin et al.^32^ observed a positive association between platelet‐to‐lymphocyte ratio (PLR) and postoperative AF after CABG. Zhang et al. developed a novel systemic inflammation score based on the integration of biomarkers including albumin, NLR, PLR, and LMR and demonstrated the association of the evaluated SIS and AF occurrence.^33^

The present study was the first to explore the relationship between LMR and mortality among AF patients. We found that the lower LMR (≤2.67) was associated with a higher risk of 28‐day, 90‐day, and 1‐year all‐cause mortality in AF patients. A PSM analysis was performed to minimize the impact of potential confounders. The major results before and after PSM were consistent in this study. However, the values of HRs on mortality after PSM were reduced compared with those before PSM, which might be due to not only the balance of baseline characteristics but also the variation of the best cutoff value after PSM. Moreover, a series of sensitivity and subgroup analyses were performed in this study to validate the robustness of our findings. An elevated monocyte count or reduced lymphocyte count might lead to a lower value of LMR. Both reduced lymphocytes and elevated monocytes are correlated with worse outcomes in terms of cardiovascular events, as reported before.^34^, ^35^ Therefore, we excluded participants with abnormal lymphocyte and monocyte counts and found that the lower LMR (≤2.67) was still correlated with a higher risk of mortality, which suggested that the LMR itself could deliver additional prognostic information, regardless of the elevated monocyte or reduced lymphocyte count. In addition, as shown in the results of the other subgroup analyses, the LMR maintained its predictive capacity despite demographic variables, comorbidities, and most of the treatment modalities. However, we found that in the subgroup of patients receiving renal replacement treatment or CABG, LMR seems not to be an independent indicator for 1‐year mortality. This might be due to CABG or renal replacement treatment, which themselves were regarded as important risk factors for AF patients, and inflammation was caused by postoperative stress response.^36^ At the same time, subgroup analysis results in the reduction of study sample size (only 73 patients remain in the subgroup of renal replacement treatment), so further researches are warranted in the future.

Despite AF is the most common form of supraventricular arrhythmia and is associated with the development of various thromboembolic complications, the exact underlying pathogenesis of AF remains only partly understood to the present day.^1^ Recently, emerging evidence suggests a significant role of inflammation in the pathogenesis of AF. Atrial electrophysiology and structural substrates could be altered by mediators of the inflammatory response, which might result in increased vulnerability to AF.^37^, ^38^ A few previous histological surveys analyzing the association between inflammation and AF have found that elevated inflammatory cell counts including lymphocytes and monocytes in human tissue samples.^11^, ^12^, ^13^, ^39^, ^40^ One recent research found a correlation between the complement system activation and lymphocyte pro‐inflammatory cytokines release with the cardiac abnormalities (conduction disturbances and atrial fibrosis/remodeling).^41^ Cluster of differentiation CD4^+^ T lymphocytes without the surface‐antigen (protein) CD28, the so‐called CD4^+^CD28^null^ T cells, are reported to be involved in chronic inflammatory processes, which might impact the development and progression of AF.^42^ Additionally, lymphopenia might indicate that the immune response is suppressed and this condition has been associated with adverse cardiac outcomes. Low relative lymphocyte count has been demonstrated to be associated with poor prognosis in patients with heart failure,^43^ acute coronary syndromes,^35^ cardiac arrest,^44^ or stable coronary heart disease.^45^ Furthermore, monocytes attach to adhesion molecules, proceeding into the sub‐endothelial space of the valve in response to locally produced cytokines such as tumor necrosis factor‐α and interleukin‐6, which might be attributed to the mechanism of AF occurrence.^46^ Abnormal changes in systemic inflammation have been related to prothrombotic indices in AF. These mechanisms might be associated with hypercoagulation, platelet activation, and endothelial dysfunction.^5^ For example, monocytes could actively bind to platelets, thus forming prothrombotic monocyte‐platelet aggregates, which might be involved in the process of atrial thrombus formation and associated with a worse prognosis in ischemic events.^15^, ^16^ The LMR integrates the clinical significance of lymphocytes and monocytes, and the underlying mechanisms might be related to the impact of low lymphocyte counts and high monocyte counts on the prognosis of AF. Additional studies are needed to investigate the exact mechanism.

Atrial fibrillation is the most common arrhythmia observed in clinical practice and a significant contributor to cardiovascular morbidity and possibly mortality.^47^ Compared with patients with sinus rhythm, patients with AF in ICU have a worse prognosis.^48^ Personalized and timely risk stratifying for each AF patient will be useful for making more precise decisions about therapeutic strategy and resource allocation. Both lymphocyte and monocyte count tests are rapid, easy, and inexpensive laboratory tests. Even under conditions without imaging or additional laboratory tests, LMR could still serve as an effective marker for quick risk assessments. In addition, in patients with AF, inflammation might be a systemic phenomenon or local process that influences the therapeutic strategies. However, to date, there is no drug that specifically targets the inflammatory pathway among AF patients. Further investigations are needed to explore the therapeutic value of LMR and find out whether anti‐inflammation therapy in AF patients with low LMR is able to ameliorate their prognosis.

Some limitations of our study should be discussed. First, data in this study were extracted from a single academic medical center in the USA, with the earliest cases from almost 20 years ago, when care may have been inconsistent with currently accepted standards. The restriction of the single‐center nature of this study might limit the generalizability of our findings. Second, The LMR was measured in AF patients only at the time of ICU entry and its dynamic alternation was not evaluated during patients’ ICU stay, which might affect the outcomes of this study. Third, in the MIMIC‐III database, values for some important variables, including types of AF, duration of AF, and AF‐related complications, were documented incompletely and not included for further analysis. Fourth, in this study, we included all ICU patients from the database. Considering the huge differences between ICU and non‐ICU patients, further studies are needed to explore the predictive value of LMR in non‐ICU patients.

## 5 CONCLUSIONS

To sum up, our study results suggested that the lower LMR (≤2.67) was correlated with a higher risk of 1‐year mortality among AF. The LMR could serve as a potential prognostic predictor of all‐cause mortality in AF patients.

## CONFLICTS OF INTEREST

Yue Yu, Suyu Wang, Pei Wang, Qiumeng Xu, Yufeng Zhang, Jian Xiao, Xiaofei Xue, Qian Yang, Wang Xi, Junnan Wang, Renhong Huang, Meiyun Liu, and Zhinong Wang report no relationships that could be construed as a conflict of interest.

## AUTHOR CONTRIBUTIONS

YY, ZW, and ML were equally responsible for the writing of the article. YY and PW conducted the statistical analyses. YY, SW, and QX participated in the study design and conduct and assisted in the writing of the article. QY, WX, RH, ML, and JW provided expert guidance in the design and conduct of this study and assisted in the writing of the article. Each author made substantial contributions to the conception or design of the work, the acquisition, analysis or interpretation of data, and drafting and final approval of the article. All authors read and approved the final article. YY, ZW, and ML conceived the study and had ultimate oversight for the design and conduct and writing of this article.

## PATIENT AND PUBLIC INVOLVEMENT

Patients and/or the public were not involved in the design, or conduct, or reporting, or dissemination plans of this research.

## PATIENT CONSENT FOR PUBLICATION

Not required.

## REPORTING CHECKLIST

The authors have completed the STROBE reporting checklist.

## Supporting information

Fig S1

Fig S2

Fig S3

Table S1‐S10

## Data Availability

Extra data can be accessed via the Dryad data repository at http://datadryad.org/ with the doi: https://doi.org/10.5061/dryad.sn02v6x4v.
