# Supplementary figures and images for: Predictive value of lymphocyte‐to‐monocyte ratio in critically Ill patients with atrial fibrillation: A propensity score matching analysis
Source: J Clin Lab Anal. 2021 Dec 30;36(2):e24217. doi: 10.1002/jcla.24217 (PMC8842191; doi:10.1002/jcla.24217)

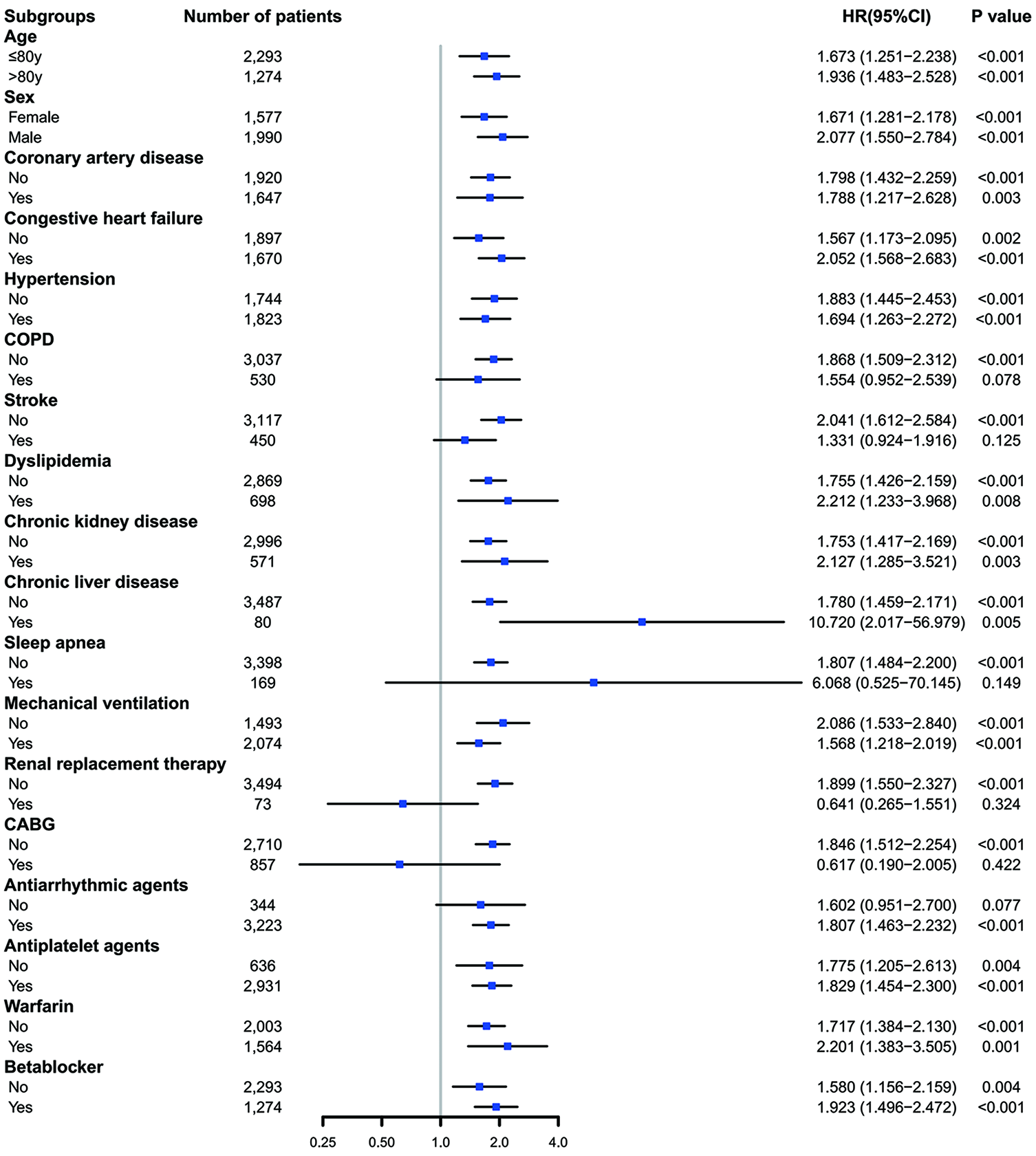

Supplement: Supplementary file 1 — Fig S1 [file JCLA-36-e24217-s001.tif]

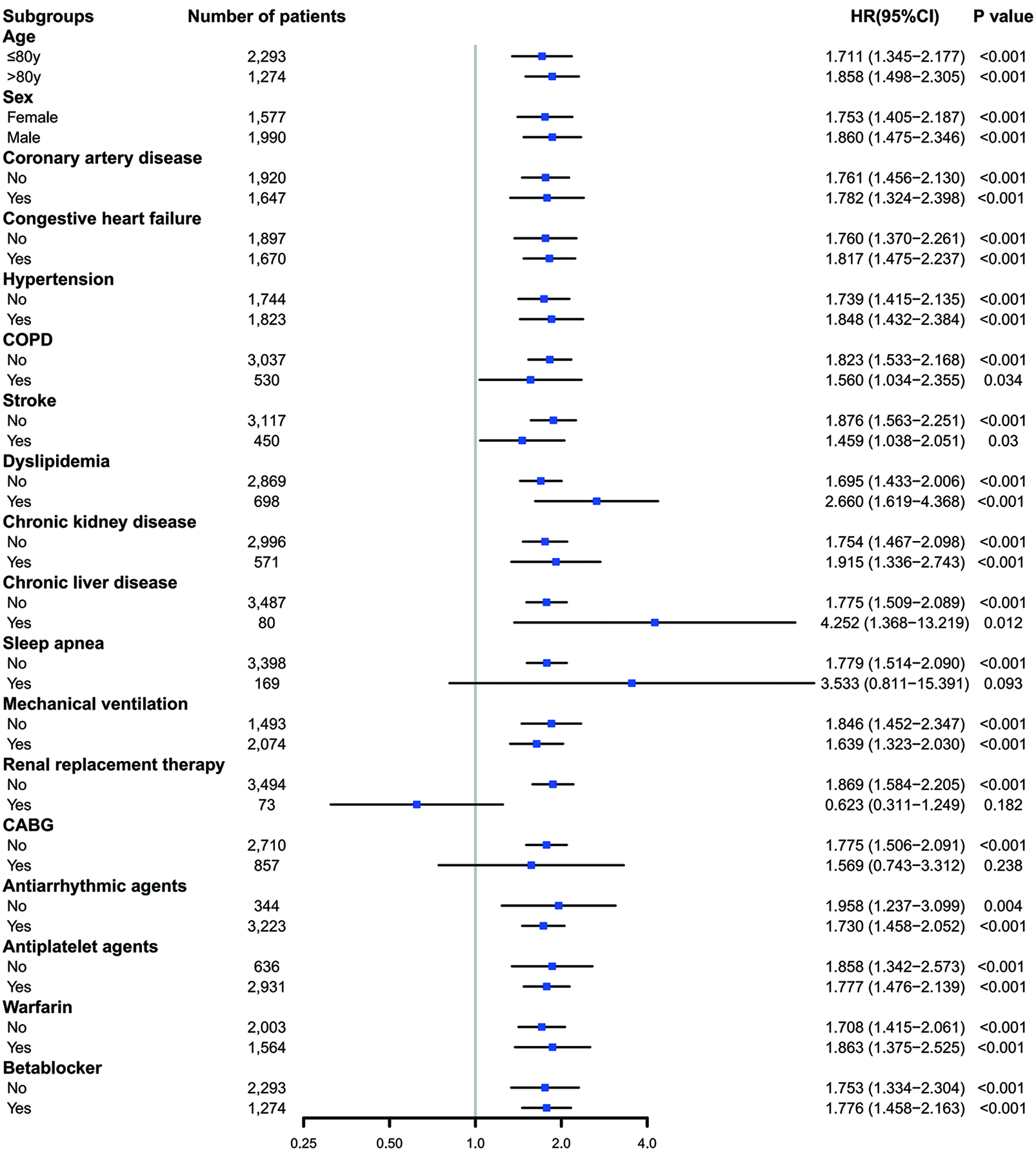

Supplement: Supplementary file 2 — Fig S2 [file JCLA-36-e24217-s004.tif]

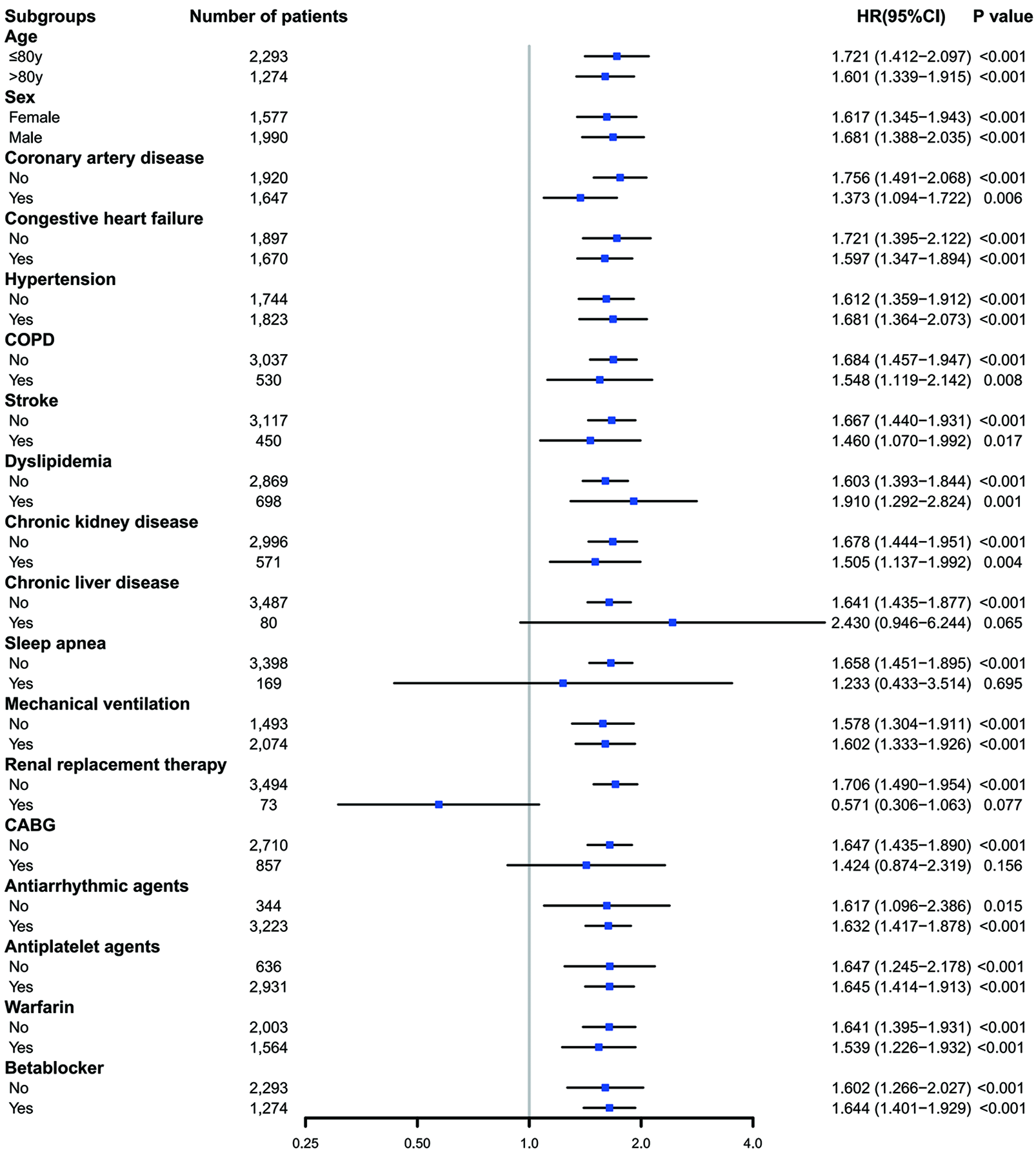

Supplement: Supplementary file 3 — Fig S3 [file JCLA-36-e24217-s003.tif]
