# Supplementary material for: Predictive value of lymphocyte‐to‐monocyte ratio in critically Ill patients with atrial fibrillation: A propensity score matching analysis
Source: J Clin Lab Anal. 2021 Dec 30;36(2):e24217. doi: 10.1002/jcla.24217 (PMC8842191; doi:10.1002/jcla.24217)
Supplement: Supplementary file 4 — Table S1‐S10 [file JCLA-36-e24217-s002.docx]

**Tables S1-10**

**TABLE S1. Univariable and multivariable Cox regression analysis for 28-day mortality before PSM.**

| **Variables** | **Unadjusted model** | | **Adjusted model 1** | | **Adjusted model 2** | |
| --- | --- | --- | --- | --- | --- | --- |
|  | **HR (95% CI)** | ***P* value** | **HR (95% CI)** | ***P* value** | **HR (95% CI)** | ***P* value** |
| LMR≤2.67 | 2.434 (2.011-2.947) | <0.001 | 2.295 (1.895-2.780) | <0.001 | 1.816 (1.494-2.208) | <0.001 |
| Age | 1.047 (1.037-1.056) | <0.001 | 1.042 (1.032-1.052) | <0.001 | 1.040 (1.030-1.050) | <0.001 |
| Sex | 0.757 (0.634-0.903) | 0.002 | 0.867 (0.725-1.037) | 0.119 | 0.999 (0.832-1.199) | 0.990 |
| CAD | 0.420 (0.345-0.512) | <0.001 |  |  | 0.884 (0.713-1.095) | 0.258 |
| Congestive heart failure | 1.591 (1.331-1.902) | <0.001 |  |  | 1.580 (1.306-1.912) | <0.001 |
| Hypertension | 0.686 (0.574-0.820) | <0.001 |  |  | 0.863 (0.704-1.058) | 0.156 |
| COPD | 1.239 (0.983-1.562) | 0.069 |  |  | 1.075 (0.850-1.360) | 0.547 |
| Stroke | 2.765 (2.263-3.378) | <0.001 |  |  | 3.228 (2.613-3.989) | <0.001 |
| TIA | 0.671 (0.334-1.350) | 0.263 |  |  |  |  |
| DM | 0.953 (0.783-1.160) | 0.630 |  |  |  |  |
| Dyslipidemia | 0.487 (0.367-0.647) | <0.001 |  |  | 0.800 (0.597-1.073) | 0.136 |
| Anemia | 0.695 (0.558-0.865) | 0.001 |  |  |  |  |
| Chronic kidney disease | 1.161 (0.923-1.460) | 0.203 |  |  | 1.034 (0.798-1.339) | 0.800 |
| Chronic liver disease | 2.590 (1.745-3.844) | <0.001 |  |  | 2.651 (1.740-4.037) | <0.001 |
| Sleep apnea | 0.314 (0.156-0.631) | 0.001 |  |  | 0.449 (0.222-0.908) | 0.026 |
| Mechanical ventilation | 1.103 (0.921-1.320) | 0.286 |  |  | 2.186 (1.807-2.645) | <0.001 |
| Renal replacement therapy | 4.928 (3.552-6.836) | <0.001 |  |  | 3.302 (2.323-4.693) | <0.001 |
| Appendage closure | 0.000 (0.000-Inf) | 0.986 |  |  | 0.000 (0.000-Inf) | 0.985 |
| CABG | 0.097 (0.059-0.160) | <0.001 |  |  | 0.125 (0.073-0.213) | <0.001 |
| Valvular surgery | 0.426 (0.190-0.952) | 0.038 |  |  | 0.607 (0.267-1.378) | 0.232 |
| Antiarrhythmic agents | 0.565 (0.442-0.724) | <0.001 |  |  | 1.224 (0.897-1.671) | 0.203 |
| Antiplatelet agents | 0.548 (0.449-0.668) | <0.001 |  |  | 0.821 (0.664-1.016) | 0.070 |
| Warfarin | 0.263 (0.210-0.331) | <0.001 |  |  | 0.306 (0.242-0.387) | <0.001 |
| Beta-blocker | 0.490 (0.409-0.587) | <0.001 |  |  | 0.638 (0.513-0.793) | <0.001 |

^†^The LMR>2.67 group was taken as the reference group.

^‡^*PSM, propensity score matching; CABG, coronary artery bypass grafting; HR, hazard ratio; CI, confidence interval; CAD, coronary artery disease; COPD, chronic obstructive pulmonary disease; HR, hazard ratio; LMR, lymphocyte-to-monocyte ratio; TIA, transient ischemic attacks*

**TABLE S2. Univariable and multivariable Cox regression analysis for 90-day mortality before PSM.**

| **Variables** | **Unadjusted model** | | **Adjusted model 1** | | **Adjusted model 2** | |
| --- | --- | --- | --- | --- | --- | --- |
|  | **HR (95% CI)** | ***P* value** | **HR (95% CI)** | ***P* value** | **HR (95% CI)** | ***P* value** |
| LMR≤2.67 | 2.312 (1.978-2.704) | <0.001 | 2.173 (1.857-2.542) | <0.001 | 1.784 (1.521-2.092) | <0.001 |
| Age | 1.052 (1.044-1.060) | <0.001 | 1.048 (1.039-1.056) | <0.001 | 1.047 (1.038-1.056) | <0.001 |
| Sex | 0.764 (0.660-0.885) | <0.001 | 0.897 (0.772-1.041) | 0.152 | 0.985 (0.846-1.146) | 0.841 |
| CAD | 0.458 (0.390-0.538) | <0.001 |  |  | 0.872 (0.730-1.041) | 0.130 |
| Congestive heart failure | 1.830 (1.575-2.126) | <0.001 |  |  | 1.637 (1.396-1.918) | <0.001 |
| Hypertension | 0.578 (0.498-0.672) | <0.001 |  |  | 0.740 (0.624-0.878) | 0.001 |
| COPD | 1.176 (0.966-1.431) | 0.105 |  |  | 0.968 (0.793-1.180) | 0.746 |
| Stroke | 2.113 (1.765-2.530) | <0.001 |  |  | 2.593 (2.149-3.130) | <0.001 |
| TIA | 1.116 (0.708-1.761) | 0.636 |  |  |  |  |
| DM | 1.028 (0.875-1.207) | 0.739 |  |  |  |  |
| Dyslipidemia | 0.460 (0.363-0.584) | <0.001 |  |  | 0.720 (0.563-0.921) | 0.009 |
| Anemia | 0.932 (0.788-1.103) | 0.411 |  |  |  |  |
| Chronic kidney disease | 1.467 (1.228-1.754) | <0.001 |  |  | 1.109 (0.906-1.358) | 0.317 |
| Chronic liver disease | 2.658 (1.901-3.717) | <0.001 |  |  | 2.990 (2.099-4.259) | <0.001 |
| Sleep apnea | 0.426 (0.260-0.700) | 0.001 |  |  | 0.589 (0.357-0.972) | 0.038 |
| Mechanical ventilation | 0.954 (0.823-1.105) | 0.529 |  |  | 1.894 (1.617-2.220) | <0.001 |
| Renal replacement therapy | 5.421 (4.094-7.178) | <0.001 |  |  | 3.827 (2.833-5.168) | <0.001 |
| Appendage closure | 0.139 (0.020-0.987) | 0.049 |  |  | 0.375 (0.053-2.684) | 0.329 |
| CABG | 0.147 (0.105-0.206) | <0.001 |  |  | 0.198 (0.137-0.286) | <0.001 |
| Valvular surgery | 0.532 (0.293-0.965) | 0.038 |  |  | 0.767 (0.418-1.407) | 0.392 |
| Antiarrhythmic agents | 0.620 (0.501-0.767) | <0.001 |  |  | 1.210 (0.925-1.583) | 0.165 |
| Antiplatelet agents | 0.561 (0.476-0.663) | <0.001 |  |  | 0.796 (0.666-0.952) | 0.012 |
| Warfarin | 0.423 (0.359-0.499) | <0.001 |  |  | 0.467 (0.393-0.555) | <0.001 |
| Beta-blocker | 0.561 (0.481-0.654) | <0.001 |  |  | 0.711 (0.590-0.856) | <0.001 |

^†^The LMR>2.67 group was taken as the reference group.

^‡^*PSM, propensity score matching; CABG, coronary artery bypass grafting; HR, hazard ratio; CI, confidence interval; CAD, coronary artery disease; COPD, chronic obstructive pulmonary disease; HR, hazard ratio; LMR, lymphocyte-to-monocyte ratio; TIA, transient ischemic attacks*

**TABLE S3. Univariable and multivariable Cox regression analysis for 1-year mortality before PSM.**

| **Variables** | **Unadjusted model** | | **Adjusted model 1** | | **Adjusted model 2** | |
| --- | --- | --- | --- | --- | --- | --- |
|  | **HR (95% CI)** | ***P* value** | **HR (95% CI)** | ***P* value** | **HR (95% CI)** | ***P* value** |
| LMR≤2.67 | 2.059 (1.809-2.343) | <0.001 | 1.950 (1.713-2.220) | <0.001 | 1.640 (1.437-1.872) | <0.001 |
| Age | 1.049 (1.042-1.056) | <0.001 | 1.045 (1.038-1.052) | <0.001 | 1.044 (1.037-1.051) | <0.001 |
| Sex | 0.787 (0.696-0.891) | <0.001 | 0.915 (0.807-1.038) | 0.168 | 0.982 (0.863-1.116) | 0.777 |
| CAD | 0.546 (0.479-0.622) | <0.001 |  |  | 0.904 (0.780-1.047) | 0.177 |
| Congestive heart failure | 1.815 (1.599-2.060) | <0.001 |  |  | 1.550 (1.356-1.773) | <0.001 |
| Hypertension | 0.581 (0.512-0.660) | <0.001 |  |  | 0.730 (0.632-0.843) | <0.001 |
| COPD | 1.268 (1.080-1.488) | 0.004 |  |  | 1.055 (0.898-1.241) | 0.514 |
| Stroke | 1.665 (1.416-1.958) | <0.001 |  |  | 2.068 (1.749-2.445) | <0.001 |
| TIA | 1.075 (0.738-1.565) | 0.707 |  |  |  |  |
| DM | 1.015 (0.886-1.162) | 0.832 |  |  |  |  |
| Dyslipidemia | 0.439 (0.360-0.535) | <0.001 |  |  | 0.631 (0.514-0.775) | <0.001 |
| Anemia | 0.967 (0.841-1.111) | 0.635 |  |  |  |  |
| Chronic kidney disease | 1.600 (1.382-1.852) | <0.001 |  |  | 1.171 (0.991-1.384) | 0.064 |
| Chronic liver disease | 2.022 (1.468-2.786) | <0.001 |  |  | 2.411 (1.727-3.365) | <0.001 |
| Sleep apnea | 0.399 (0.262-0.610) | <0.001 |  |  | 0.537 (0.351-0.823) | 0.004 |
| Mechanical ventilation | 0.877 (0.774-0.993) | 0.038 |  |  | 1.663 (1.453-1.902) | <0.001 |
| Renal replacement therapy | 5.162 (3.987-6.682) | <0.001 |  |  | 3.877 (2.947-5.100) | <0.001 |
| Appendage closure | 0.161 (0.040-0.647) | 0.010 |  |  | 0.382 (0.095-1.538) | 0.176 |
| CABG | 0.230 (0.181-0.291) | <0.001 |  |  | 0.292 (0.223-0.382) | <0.001 |
| Valvular surgery | 0.500 (0.295-0.847) | 0.010 |  |  | 0.709 (0.415-1.211) | 0.208 |
| Antiarrhythmic agents | 0.723 (0.599-0.873) | 0.001 |  |  | 1.261 (0.996-1.596) | 0.054 |
| Antiplatelet agents | 0.629 (0.544-0.727) | <0.001 |  |  | 0.838 (0.718-0.978) | 0.025 |
| Warfarin | 0.546 (0.479-0.623) | <0.001 |  |  | 0.581 (0.506-0.666) | <0.001 |
| Beta-blocker | 0.651 (0.570-0.743) | <0.001 |  |  | 0.770 (0.656-0.903) | 0.001 |

^†^The LMR>2.67 group was taken as the reference group.

^‡^*PSM, propensity score matching; CABG, coronary artery bypass grafting; HR, hazard ratio; CI, confidence interval; CAD, coronary artery disease; COPD, chronic obstructive pulmonary disease; HR, hazard ratio; LMR, lymphocyte-to-monocyte ratio; TIA, transient ischemic attacks*

**TABLE S4. Univariable and multivariable Cox regression analysis for 28-day mortality after PSM.**

| **Variables** | **Unadjusted model** | | **Adjusted model 1** | | **Adjusted model 2** | |
| --- | --- | --- | --- | --- | --- | --- |
|  | **HR (95% CI)** | ***P* value** | **HR (95% CI)** | ***P* value** | **HR (95% CI)** | ***P* value** |
| LMR≤2.67 | 1.403 (1.111-1.771) | 0.004 | 1.396 (1.106-1.763) | 0.005 | 1.447 (1.145-1.830) | 0.002 |
| Age | 1.043 (1.031-1.056) | <0.001 | 1.040 (1.027-1.053) | <0.001 | 1.043 (1.029-1.057) | <0.001 |
| Sex | 0.625 (0.496-0.788) | <0.001 | 0.747 (0.589-0.946) | 0.015 | 0.896 (0.705-1.140) | 0.373 |
| CAD | 0.378 (0.290-0.494) | <0.001 |  |  | 0.723 (0.536-0.976) | 0.034 |
| Congestive heart failure | 1.410 (1.118-1.779) | 0.004 |  |  | 1.498 (1.167-1.923) | 0.002 |
| Hypertension | 0.731 (0.580-0.922) | 0.008 |  |  | 0.888 (0.677-1.166) | 0.393 |
| COPD | 1.186 (0.872-1.613) | 0.276 |  |  | 1.216 (0.887-1.668) | 0.224 |
| Stroke | 2.760 (2.129-3.578) | <0.001 |  |  | 3.081 (2.347-4.046) | <0.001 |
| TIA | 0.822 (0.366-1.844) | 0.634 |  |  |  |  |
| DM | 0.875 (0.672-1.140) | 0.324 |  |  |  |  |
| Dyslipidemia | 0.674 (0.481-0.944) | 0.022 |  |  | 0.988 (0.694-1.406) | 0.948 |
| Anemia | 0.693 (0.520-0.922) | 0.012 |  |  |  |  |
| Chronic kidney disease | 1.047 (0.766-1.430) | 0.774 |  |  | 1.003 (0.702-1.433) | 0.987 |
| Chronic liver disease | 2.421 (1.415-4.142) | 0.001 |  |  | 2.938 (1.657-5.207) | <0.001 |
| Sleep apnea | 0.192 (0.062-0.600) | 0.005 |  |  | 0.302 (0.097-0.947) | 0.040 |
| Mechanical ventilation | 1.089 (0.863-1.374) | 0.473 |  |  | 2.061 (1.602-2.653) | <0.001 |
| Renal replacement therapy | 6.475 (4.260-9.842) | <0.001 |  |  | 6.561 (4.132-10.420) | <0.001 |
| Appendage closure | 0.000 (0.000-Inf) | 0.991 |  |  | 0.000 (0.000-Inf) | 0.990 |
| CABG | 0.150 (0.086-0.262) | <0.001 |  |  | 0.210 (0.113-0.389) | <0.001 |
| Valvular surgery | 0.606 (0.226-1.625) | 0.319 |  |  | 0.445 (0.157-1.259) | 0.127 |
| Antiarrhythmic agents | 0.648 (0.459-0.915) | 0.014 |  |  | 1.470 (0.961-2.248) | 0.075 |
| Antiplatelet agents | 0.577 (0.446-0.746) | <0.001 |  |  | 0.729 (0.553-0.961) | 0.025 |
| Warfarin | 0.276 (0.205-0.371) | <0.001 |  |  | 0.296 (0.218-0.402) | <0.001 |
| Beta-blocker | 0.518 (0.409-0.656) | <0.001 |  |  | 0.632 (0.477-0.838) | 0.001 |

^†^The LMR>2.67 group was taken as the reference group.

^‡^*PSM, propensity score matching; CABG, coronary artery bypass grafting; HR, hazard ratio; CI, confidence interval; CAD, coronary artery disease; COPD, chronic obstructive pulmonary disease; HR, hazard ratio; LMR, lymphocyte-to-monocyte ratio; TIA, transient ischemic attacks*

**TABLE S5. Univariable and multivariable Cox regression analysis for 90-day mortality after PSM**

| **Variables** | **Unadjusted model** | | **Adjusted model 1** | | **Adjusted model 2** | |
| --- | --- | --- | --- | --- | --- | --- |
|  | **HR (95% CI)** | ***P* value** | **HR (95% CI)** | ***P* value** | **HR (95% CI)** | ***P* value** |
| LMR≤2.67 | 1.341 (1.113-1.617) | 0.002 | 1.344 (1.115-1.619) | 0.002 | 1.416 (1.174-1.708) | <0.001 |
| Age | 1.047 (1.037-1.057) | <0.001 | 1.045 (1.034-1.055) | <0.001 | 1.046 (1.035-1.058) | <0.001 |
| Sex | 0.697 (0.579-0.839) | <0.001 | 0.851 (0.704-1.028) | 0.095 | 0.950 (0.784-1.153) | 0.606 |
| CAD | 0.474 (0.387-0.580) | <0.001 |  |  | 0.809 (0.644-1.017) | 0.069 |
| Congestive heart failure | 1.727 (1.428-2.089) | <0.001 |  |  | 1.622 (1.325-1.986) | <0.001 |
| Hypertension | 0.591 (0.489-0.714) | <0.001 |  |  | 0.733 (0.589-0.912) | 0.005 |
| COPD | 1.058 (0.818-1.367) | 0.668 |  |  | 1.000 (0.770-1.300) | 0.998 |
| Stroke | 2.113 (1.686-2.648) | <0.001 |  |  | 2.535 (2.005-3.204) | <0.001 |
| TIA | 1.358 (0.812-2.273) | 0.243 |  |  |  |  |
| DM | 1.016 (0.828-1.247) | 0.880 |  |  |  |  |
| Dyslipidemia | 0.632 (0.480-0.833) | 0.001 |  |  | 0.847 (0.636-1.129) | 0.258 |
| Anemia | 1.017 (0.826-1.252) | 0.877 |  |  |  |  |
| Chronic kidney disease | 1.461 (1.163-1.834) | 0.001 |  |  | 1.145 (0.881-1.489) | 0.311 |
| Chronic liver disease | 2.527 (1.630-3.916) | <0.001 |  |  | 3.162 (1.989-5.028) | <0.001 |
| Sleep apnea | 0.367 (0.190-0.711) | 0.003 |  |  | 0.551 (0.283-1.072) | 0.079 |
| Mechanical ventilation | 0.915 (0.760-1.102) | 0.350 |  |  | 1.709 (1.394-2.094) | <0.001 |
| Renal replacement therapy | 7.131 (4.992-10.187) | <0.001 |  |  | 7.053 (4.778-10.413) | <0.001 |
| Appendage closure | 0.325 (0.046-2.311) | 0.261 |  |  | 0.541 (0.075-3.893) | 0.542 |
| CABG | 0.181 (0.121-0.271) | <0.001 |  |  | 0.247 (0.157-0.389) | <0.001 |
| Valvular surgery | 0.677 (0.321-1.428) | 0.306 |  |  | 0.595 (0.272-1.300) | 0.193 |
| Antiarrhythmic agents | 0.687 (0.517-0.913) | 0.010 |  |  | 1.381 (0.969-1.970) | 0.074 |
| Antiplatelet agents | 0.613 (0.497-0.757) | <0.001 |  |  | 0.743 (0.593-0.933) | 0.010 |
| Warfarin | 0.460 (0.374-0.565) | <0.001 |  |  | 0.457 (0.369-0.567) | <0.001 |
| Beta-blocker | 0.616 (0.506-0.749) | <0.001 |  |  | 0.742 (0.587-0.939) | 0.013 |

^†^The LMR>2.67 group was taken as the reference group.

^‡^*PSM, propensity score matching; CABG, coronary artery bypass grafting; HR, hazard ratio; CI, confidence interval; CAD, coronary artery disease; COPD, chronic obstructive pulmonary disease; HR, hazard ratio; LMR, lymphocyte-to-monocyte ratio; TIA, transient ischemic attacks*

**TABLE S6. Univariable and multivariable Cox regression analysis for 1-year mortality after PSM.**

| **Variables** | **Unadjusted model** | | **Adjusted model 1** | | **Adjusted model 2** | |
| --- | --- | --- | --- | --- | --- | --- |
|  | **HR (95% CI)** | ***P* value** | **HR (95% CI)** | ***P* value** | **HR (95% CI)** | ***P* value** |
| LMR≤2.67 | 1.223 (1.047-1.429) | 0.011 | 1.217 (1.042-1.422) | 0.013 | 1.279 (1.094-1.495) | 0.002 |
| Age | 1.044 (1.036-1.053) | <0.001 | 1.042 (1.033-1.051) | <0.001 | 1.043 (1.033-1.052) | <0.001 |
| Sex | 0.706 (0.604-0.824) | <0.001 | 0.851 (0.726-0.998) | 0.047 | 0.921 (0.783-1.083) | 0.319 |
| CAD | 0.572 (0.486-0.674) | <0.001 |  |  | 0.856 (0.710-1.031) | 0.102 |
| Congestive heart failure | 1.751 (1.492-2.054) | <0.001 |  |  | 1.570 (1.325-1.862) | <0.001 |
| Hypertension | 0.590 (0.504-0.692) | <0.001 |  |  | 0.715 (0.596-0.857) | <0.001 |
| COPD | 1.103 (0.896-1.359) | 0.355 |  |  | 1.031 (0.835-1.274) | 0.776 |
| Stroke | 1.686 (1.378-2.061) | <0.001 |  |  | 2.045 (1.663-2.515) | <0.001 |
| TIA | 1.354 (0.893-2.054) | 0.153 |  |  |  |  |
| DM | 0.973 (0.819-1.156) | 0.757 |  |  |  |  |
| Dyslipidemia | 0.577 (0.458-0.728) | <0.001 |  |  | 0.719 (0.565-0.914) | 0.007 |
| Anemia | 1.030 (0.867-1.223) | 0.739 |  |  |  |  |
| Chronic kidney disease | 1.603 (1.332-1.930) | <0.001 |  |  | 1.235 (0.996-1.531) | 0.054 |
| Chronic liver disease | 1.921 (1.277-2.888) | 0.002 |  |  | 2.438 (1.593-3.732) | <0.001 |
| Sleep apnea | 0.381 (0.224-0.646) | <0.001 |  |  | 0.548 (0.322-0.936) | 0.027 |
| Mechanical ventilation | 0.861 (0.737-1.005) | 0.058 |  |  | 1.516 (1.278-1.799) | <0.001 |
| Renal replacement therapy | 6.833 (4.889-9.551) | <0.001 |  |  | 6.740 (4.706-9.654) | <0.001 |
| Appendage closure | 0.380 (0.095-1.525) | 0.172 |  |  | 0.577 (0.143-2.333) | 0.441 |
| CABG | 0.261 (0.195-0.351) | <0.001 |  |  | 0.340 (0.242-0.477) | <0.001 |
| Valvular surgery | 0.633 (0.328-1.222) | 0.173 |  |  | 0.636 (0.324-1.250) | 0.189 |
| Antiarrhythmic agents | 0.788 (0.615-1.010) | 0.060 |  |  | 1.375 (1.009-1.875) | 0.044 |
| Antiplatelet agents | 0.699 (0.583-0.838) | <0.001 |  |  | 0.805 (0.662-0.979) | 0.030 |
| Warfarin | 0.603 (0.512-0.710) | <0.001 |  |  | 0.579 (0.488-0.687) | <0.001 |
| Beta-blocker | 0.727 (0.615-0.861) | <0.001 |  |  | 0.837 (0.684-1.024) | 0.084 |

^†^The LMR>2.67 group was taken as the reference group.

^‡^*PSM, propensity score matching; CABG, coronary artery bypass grafting; HR, hazard ratio; CI, confidence interval; CAD, coronary artery disease; COPD, chronic obstructive pulmonary disease; HR, hazard ratio; LMR, lymphocyte-to-monocyte ratio; TIA, transient ischemic attacks*

**TABLE S7. Univariable and multivariable Cox regression analysis for 28-day mortality for patients with normal lymphocyte and monocyte counts.**

| **Variables** | **Unadjusted model** | | **Adjusted model 1** | | **Adjusted model 2** | |
| --- | --- | --- | --- | --- | --- | --- |
|  | **HR (95% CI)** | ***P* value** | **HR (95% CI)** | ***P* value** | **HR (95% CI)** | ***P* value** |
| LMR≤2.67 | 2.215 (1.730-2.836) | <0.001 | 2.095 (1.635-2.685) | <0.001 | 1.755 (1.360-2.266) | <0.001 |
| Age | 1.047 (1.034-1.061) | <0.001 | 1.041 (1.028-1.055) | <0.001 | 1.039 (1.025-1.054) | <0.001 |
| Sex | 0.645 (0.503-0.827) | 0.001 | 0.769 (0.596-0.991) | 0.043 | 0.921 (0.710-1.196) | 0.539 |
| CAD | 0.471 (0.361-0.615) | <0.001 |  |  | 0.880 (0.653-1.187) | 0.403 |
| Congestive heart failure | 1.516 (1.182-1.944) | 0.001 |  |  | 1.443 (1.097-1.898) | 0.009 |
| Hypertension | 0.731 (0.570-0.937) | 0.013 |  |  | 0.843 (0.631-1.125) | 0.245 |
| COPD | 1.206 (0.861-1.691) | 0.276 |  |  | 1.043 (0.738-1.474) | 0.811 |
| Stroke | 3.155 (2.422-4.111) | <0.001 |  |  | 3.399 (2.568-4.498) | <0.001 |
| TIA | 0.537 (0.200-1.442) | 0.218 |  |  |  |  |
| DM | 0.908 (0.687-1.199) | 0.494 |  |  |  |  |
| Dyslipidemia | 0.642 (0.454-0.906) | 0.012 |  |  | 0.906 (0.631-1.299) | 0.590 |
| Anemia | 0.780 (0.581-1.048) | 0.099 |  |  |  |  |
| Chronic kidney disease | 1.185 (0.861-1.631) | 0.298 |  |  | 0.888 (0.613-1.286) | 0.529 |
| Chronic liver disease | 1.445 (0.643-3.247) | 0.373 |  |  | 1.821 (0.790-4.196) | 0.159 |
| Sleep apnea | 0.423 (0.188-0.950) | 0.037 |  |  | 0.439 (0.192-1.003) | 0.051 |
| Mechanical ventilation | 1.192 (0.927-1.532) | 0.172 |  |  | 2.414 (1.851-3.148) | <0.001 |
| Renal replacement therapy | 7.394 (4.677-11.690) | <0.001 |  |  | 7.688 (4.584-12.893) | <0.001 |
| Appendage closure | 0.000 (0.000-Inf) | 0.991 |  |  | 0.000 (0.000-Inf) | 0.990 |
| CABG | 0.115 (0.061-0.216) | <0.001 |  |  | 0.130 (0.066-0.258) | <0.001 |
| Valvular surgery | 0.553 (0.206-1.484) | 0.239 |  |  | 0.773 (0.282-2.117) | 0.617 |
| Antiarrhythmic agents | 0.649 (0.454-0.928) | 0.018 |  |  | 1.839 (1.177-2.873) | 0.007 |
| Antiplatelet agents | 0.574 (0.433-0.762) | <0.001 |  |  | 0.769 (0.565-1.047) | 0.095 |
| Warfarin | 0.310 (0.229-0.419) | <0.001 |  |  | 0.353 (0.258-0.481) | <0.001 |
| Beta-blocker | 0.511 (0.396-0.659) | <0.001 |  |  | 0.511 (0.377-0.692) | <0.001 |

^†^The LMR>2.67 group was taken as the reference group.

^‡^*CABG, coronary artery bypass grafting; HR, hazard ratio; CI, confidence interval; CAD, coronary artery disease; COPD, chronic obstructive pulmonary disease; HR, hazard ratio; LMR, lymphocyte-to-monocyte ratio; TIA, transient ischemic attacks*

**TABLE S8. Univariable and multivariable Cox regression analysis for 90-day mortality for patients with normal lymphocyte and monocyte counts.**

| **Variables** | **Unadjusted model** | | **Adjusted model 1** | | **Adjusted model 2** | |
| --- | --- | --- | --- | --- | --- | --- |
|  | **HR (95% CI)** | ***P* value** | **HR (95% CI)** | ***P* value** | **HR (95% CI)** | ***P* value** |
| LMR≤2.67 | 1.973 (1.601-2.432) | <0.001 | 1.852 (1.501-2.284) | <0.001 | 1.548 (1.249-1.920) | <0.001 |
| Age | 1.054 (1.042-1.066) | <0.001 | 1.049 (1.037-1.061) | <0.001 | 1.045 (1.033-1.058) | <0.001 |
| Sex | 0.655 (0.531-0.808) | <0.001 | 0.806 (0.650-0.999) | 0.049 | 0.904 (0.725-1.127) | 0.369 |
| CAD | 0.513 (0.411-0.641) | <0.001 |  |  | 0.968 (0.756-1.238) | 0.795 |
| Congestive heart failure | 1.822 (1.472-2.255) | <0.001 |  |  | 1.602 (1.271-2.020) | <0.001 |
| Hypertension | 0.637 (0.516-0.787) | <0.001 |  |  | 0.752 (0.590-0.960) | 0.022 |
| COPD | 1.113 (0.829-1.494) | 0.477 |  |  | 0.960 (0.711-1.295) | 0.787 |
| Stroke | 2.452 (1.935-3.108) | <0.001 |  |  | 2.881 (2.245-3.698) | <0.001 |
| TIA | 0.970 (0.517-1.818) | 0.923 |  |  |  |  |
| DM | 0.902 (0.713-1.142) | 0.392 |  |  |  |  |
| Dyslipidemia | 0.548 (0.403-0.745) | <0.001 |  |  | 0.752 (0.547-1.035) | 0.080 |
| Anemia | 0.971 (0.767-1.230) | 0.810 |  |  |  |  |
| Chronic kidney disease | 1.455 (1.127-1.877) | 0.004 |  |  | 0.981 (0.728-1.322) | 0.900 |
| Chronic liver disease | 1.998 (1.096-3.643) | 0.024 |  |  | 2.655 (1.426-4.943) | 0.002 |
| Sleep apnea | 0.606 (0.341-1.078) | 0.088 |  |  | 0.615 (0.340-1.113) | 0.109 |
| Mechanical ventilation | 1.013 (0.821-1.250) | 0.902 |  |  | 2.146 (1.716-2.685) | <0.001 |
| Renal replacement therapy | 7.510 (4.951-11.394) | <0.001 |  |  | 7.409 (4.652-11.798) | <0.001 |
| Appendage closure | 0.361 (0.051-2.574) | 0.310 |  |  | 0.545 (0.075-3.936) | 0.547 |
| CABG | 0.120 (0.071-0.201) | <0.001 |  |  | 0.135 (0.077-0.236) | <0.001 |
| Valvular surgery | 0.489 (0.202-1.182) | 0.112 |  |  | 0.752 (0.307-1.844) | 0.534 |
| Antiarrhythmic agents | 0.691 (0.507-0.942) | 0.019 |  |  | 1.745 (1.181-2.577) | 0.005 |
| Antiplatelet agents | 0.609 (0.477-0.776) | <0.001 |  |  | 0.811 (0.622-1.057) | 0.121 |
| Warfarin | 0.430 (0.341-0.543) | <0.001 |  |  | 0.458 (0.359-0.584) | <0.001 |
| Beta-blocker | 0.580 (0.465-0.723) | <0.001 |  |  | 0.560 (0.430-0.729) | <0.001 |

^†^The LMR>2.67 group was taken as the reference group.

^‡^*CABG, coronary artery bypass grafting; HR, hazard ratio; CI, confidence interval; CAD, coronary artery disease; COPD, chronic obstructive pulmonary disease; HR, hazard ratio; LMR, lymphocyte-to-monocyte ratio; TIA, transient ischemic attacks*

**TABLE S9. Univariable and multivariable Cox regression analysis for 1-year mortality for patients with normal lymphocyte and monocyte counts.**

| **Variables** | **Unadjusted model** | | **Adjusted model 1** | | **Adjusted model 2** | |
| --- | --- | --- | --- | --- | --- | --- |
|  | **HR (95% CI)** | ***P* value** | **HR (95% CI)** | ***P* value** | **HR (95% CI)** | ***P* value** |
| LMR≤2.67 | 1.781 (1.496-2.121) | <0.001 | 1.674 (1.405-1.994) | <0.001 | 1.442 (1.205-1.724) | <0.001 |
| Age | 1.049 (1.039-1.059) | <0.001 | 1.045 (1.035-1.055) | <0.001 | 1.040 (1.030-1.050) | <0.001 |
| Sex | 0.710 (0.597-0.845) | <0.001 | 0.862 (0.721-1.030) | 0.102 | 0.907 (0.755-1.089) | 0.295 |
| CAD | 0.611 (0.511-0.732) | <0.001 |  |  | 1.004 (0.819-1.230) | 0.969 |
| Congestive heart failure | 1.816 (1.521-2.170) | <0.001 |  |  | 1.541 (1.274-1.865) | <0.001 |
| Hypertension | 0.592 (0.496-0.706) | <0.001 |  |  | 0.716 (0.585-0.877) | 0.001 |
| COPD | 1.184 (0.934-1.499) | 0.162 |  |  | 1.028 (0.808-1.307) | 0.822 |
| Stroke | 1.778 (1.436-2.200) | <0.001 |  |  | 2.197 (1.760-2.742) | <0.001 |
| TIA | 1.036 (0.639-1.680) | 0.886 |  |  |  |  |
| DM | 0.893 (0.735-1.084) | 0.253 |  |  |  |  |
| Dyslipidemia | 0.500 (0.387-0.647) | <0.001 |  |  | 0.643 (0.493-0.839) | 0.001 |
| Anemia | 0.974 (0.803-1.183) | 0.793 |  |  |  |  |
| Chronic kidney disease | 1.700 (1.389-2.081) | <0.001 |  |  | 1.187 (0.938-1.502) | 0.154 |
| Chronic liver disease | 1.554 (0.896-2.696) | 0.117 |  |  | 2.103 (1.194-3.705) | 0.010 |
| Sleep apnea | 0.510 (0.305-0.852) | 0.010 |  |  | 0.532 (0.314-0.902) | 0.019 |
| Mechanical ventilation | 0.915 (0.769-1.089) | 0.318 |  |  | 1.832 (1.518-2.211) | <0.001 |
| Renal replacement therapy | 6.497 (4.369-9.662) | <0.001 |  |  | 5.600 (3.629-8.642) | <0.001 |
| Appendage closure | 0.420 (0.105-1.683) | 0.220 |  |  | 0.575 (0.142-2.329) | 0.438 |
| CABG | 0.222 (0.159-0.311) | <0.001 |  |  | 0.245 (0.168-0.359) | <0.001 |
| Valvular surgery | 0.496 (0.235-1.045) | 0.065 |  |  | 0.731 (0.343-1.557) | 0.417 |
| Antiarrhythmic agents | 0.761 (0.583-0.992) | 0.044 |  |  | 1.525 (1.091-2.131) | 0.014 |
| Antiplatelet agents | 0.682 (0.554-0.841) | <0.001 |  |  | 0.872 (0.696-1.093) | 0.236 |
| Warfarin | 0.570 (0.475-0.684) | <0.001 |  |  | 0.581 (0.480-0.704) | <0.001 |
| Beta-blocker | 0.677 (0.561-0.816) | <0.001 |  |  | 0.665 (0.530-0.835) | <0.001 |

^†^The LMR>2.67 group was taken as the reference group.

^‡^*CABG, coronary artery bypass grafting; HR, hazard ratio; CI, confidence interval; CAD, coronary artery disease; COPD, chronic obstructive pulmonary disease; HR, hazard ratio; LMR, lymphocyte-to-monocyte ratio; TIA, transient ischemic attacks*

**TABLE S10. HR for LMR as a continuous variable.**

|  | **28-day mortality** | | | **90-day mortality** | | | **1-year mortality** | | |
| --- | --- | --- | --- | --- | --- | --- | --- | --- | --- |
|  | **Piece-wise LMR** | **HR (95%CI)** | ***P* value** | **Piece-wise LMR** | **HR (95%CI)** | ***P* value** | **Piece-wise LMR** | **HR (95%CI)** | ***P* value** |
| **Cox regression model** | - | 0.863 (0.817 -0.911) | <0.001 | - | 0.878 (0.840 -0.917) | <0.001 | - | 0.895 (0.864 -0.927) | <0.001 |
| **Two piece-wise Cox regression model** | LMR<5.63 | 0.835 (0.780 -0.894) | <0.001 | LMR<5.33 | 0.811 (0.766 -0.859) | <0.001 | LMR<5.50 | 0.833 (0.795 -0.873) | <0.001 |
|  | LMR>5.63 | 0.959 (0.843 -1.090) | 0.518 | LMR>5.33 | 1.021 (0.959 -1.086) | 0.519 | LMR>5.50 | 1.019 (0.969 -1.072) | 0.464 |
| **Logarithm likelihood ratio test*** | - | - | 0.133 | - | - | <0.001 | - | - | <0.001 |

^†^All HRs were adjusted for age, gender, CAD, congestive heart failure, hypertension, COPD, stroke, dyslipidemia, chronic kidney disease, chronic liver disease, sleep apnea, mechanical ventilation, renal replacement treatment, appendage closure, CABG, valvular surgery, antiarrhythmic, antiplatelet agents, warfarin, Beta-blocker.

^‡^logarithm likelihood ratio test was used to compare the Cox regression model with the two piece-wise Cox regression model.

^§^*LMR, lymphocyte-to-monocyte ratio;* CABG, coronary artery bypass grafting; *HR, hazard ratio;* CI, confidential interval; COPD, chronic obstructive pulmonary disease; *CAD, coronary artery disease*
